# The composition of the dental pellicle: an updated literature review

**DOI:** 10.3389/froh.2023.1260442

**Published:** 2023-10-12

**Authors:** Joachim Enax, Bernhard Ganss, Bennett T. Amaechi, Erik Schulze zur Wiesche, Frederic Meyer

**Affiliations:** ^1^ Research Department, Dr. Kurt Wolff GmbH & Co. KG, Bielefeld, Germany; ^2^ Faculty of Dentistry, University of Toronto, Toronto, ON, Canada; ^3^ Department of Comprehensive Dentistry, School of Dentistry, University of Texas Health San Antonio, San Antonio, TX, United States

**Keywords:** dental pellicle, proteins, saliva, biofilm, teeth, hydroxyapatite

## Abstract

**Background:**

The dental pellicle is a thin layer of up to several hundred nm in thickness, covering the tooth surface. It is known to protect the teeth from acid attacks through its selective permeability and it is involved in the remineralization process of the teeth. It functions also as binding site and source of nutrients for bacteria and conditioning biofilm (foundation) for dental plaque formation.

**Methods:**

For this updated literature review, the PubMed database was searched for the dental pellicle and its composition.

**Results:**

The dental pellicle has been analyzed in the past years with various state-of-the art analytic techniques such as high-resolution microscopic techniques (e.g., scanning electron microscopy, atomic force microscopy), spectrophotometry, mass spectrometry, affinity chromatography, enzyme-linked immunosorbent assays (ELISA), and blotting-techniques (e.g., western blot). It consists of several different amino acids, proteins, and proteolytic protein fragments. Some studies also investigated other compounds of the pellicle, mainly fatty acids, and carbohydrates.

**Conclusions:**

The dental pellicle is composed mainly of different proteins, but also fatty acids, and carbohydrates. Analysis with state-of-the-art analytical techniques have uncovered mainly acidic proline-rich proteins, amylase, cystatin, immunoglobulins, lysozyme, and mucins as main proteins of the dental pellicle. The pellicle has protective properties for the teeth. Further research is necessary to gain more knowledge about the role of the pellicle in the tooth remineralization process.

## 1. Introduction

The dental pellicle (from the latin word “pellicula” which means membrane or thin film) is a very thin biofilm coating the surfaces of human teeth and oral mucosa. It mainly consists of proteins, amino and fatty acids, glycoproteins, carbohydrates, lipids, and other compounds found in saliva, but also those derived from microorganisms, such as bacteria and fungi can be found here (1–16). The dental pellicle has a zeta-potential of −15 to −30 mV. However, with saliva the net-surface changes to −8 to −14 mV (17). These data indicate that the pellicle is anionic (3–6). Jensen et al. have shown that the composition of the dental pellicle is heterogenous among subjects (18). They isolated all proteins found in saliva using anionic and cationic discontinuous polyacrylamide gel electrophoresis. Intact proteins and their proteolytic fragments can be found here. Mainly amylase, acidic proline-rich proteins, mucins (MUC5B and MUC7), cystatins as well as proteolytically derived peptides, amylase, lysozymes, and glycosyltransferase can be found in the pellicle. Acidic phosphoproteins, neutral and basic histatins selectively adsorb to hydroxyapatite (2, 3, 18, 19). Interestingly, no difference in the pellicle composition could be observed between healthy individuals and those with active caries. Here, different mass spectrometry-based techniques were used to investigate the dental pellicle (20). Vacca-Smith et al. found in their in-situ study similar proteins at different time-points during pellicle formation. The amount of proteins was also comparable between time-points of collection (21). The morphological analysis revealed a diameter of adsorbed proteins of 15 ( ± 3) nm (17). The thickness of the pellicle differed in various studies between 2 ( ± 0.5) nm, 18 nm (22), and 100 nm to 1,000 nm depending on the location of the tooth (3, 17). However, high sugary diet changed the pattern of the dental pellicle composition (21). Adhesins from microorganisms can bind to the salivary proteins found in the pellicle. Interestingly, Streptococci bind to amylase and salivary agglutinin glycoprotein (1, 23). However, bacteria are not part of the dental pellicle. Nevertheless, components from bacteria can be found in this protein-containing layer (2).

The pellicle functions as lubrication layer and has protective properties for the dentition. Lubarsky et al. have reported that the pellicle can help defending the teeth from acidic attacks due to its selective permeability. The pellicle proteins can be found in small cracks in enamel as well. This might influence the mechanical properties of the teeth (24). However, full protection of the teeth by the pellicle is not possible (25). Hara et al. showed that the dental pellicle formed after 2 h can protect teeth from demineralization due to erosive challenge for 10 min, but not a longer period. Dentin could not be protected (26). Amaechi et al. confirmed these results for the 1 h enamel pellicle (16). However, *in vivo* the dental pellicle will be colonized within seconds to minutes (27). Protective characteristics of the pellicle will differ between the individuals and their respective pelliclecomposition.

The formation of the pellicle is a specific and non-random process (3). The binding of salivary proteins, especially aPRPs, statherin, and histatins, are the first proteins that adsorb to hydroxyapatite of enamel (3, 5). More than 100 different proteins can be found in the pellicle (3, 6). Trautmann et al. found a 10 times higher number of proteins from the pellicle compared to the previously published studies: of the 1,188 identified proteins, 68 proteins were found in all individuals (caries-active and healthy) investigated in the study (9, 10). The same research group investigated the composition of pellicle with saliva of the respective subjects. 498 proteins in the dental pellicle, and 1,032 proteins in the saliva could be identified. Additionally, pellicle formation relies on selective adsorption (10). Here, nano-liquid-chromatography-high resolution-mass-spectrometry/mass-spectrometry (LC-HR-MS/MS) techniques were used for the identification of the pellicle composition. The formation of the pellicle takes about 30 to 90 min (10). It has been shown that acids can remove the outer globular layer of the pellicle, but the basal layer stays intact. Acid resistance is mediated by statherins and mucins (3). When discussing protective properties, about 8% of the pellicle-bound proteins have additional antibacterial properties: cystatins, lysozyme, myeloperoxidase, and histatins (5). Delvar et al. have shown that the use of carboxymethylcellulose helps to improve the protective properties of the pellicle (28). The same could be observed for chitosan, which adsorbs on top of the pellicle. Interestingly, individual variations seem to be important in the protective action of the dental pellicle. This was observed by Bruvo et al. with the help of different methods, such as surface microhardness, sodium dodecyl sulfate—poly crylamide gel electrophoresis (SDS-PAGE), and high-pressure liquid chromatography (HPLC) (29). Another study investigated the protective properties of calcium ions, which can also be incorporated into the pellicle (30). Increased calcium concentration in saliva, and in the pellicle, weakens the electrostatic interaction between salivary proteins and the enamel surface. At the same time, thickness and viscoelasticity of the pellicle are increased (31). The density and mechanical properties increase from the outer to the inner layer (22). This was measured by nanoindentation. Using ellipsometry and transmission electron microscopy (TEM), Güth-Thiel et al. observed that the pellicle formation can be separated into a rapid formation-phase within the first minutes after tooth cleaning, and a slowpellicle formation-phase with only minor changes in composition and thickness between 30 and 120 min (11).

While several studies have looked at different aspects of the enamel pellicle, a conclusive and state-of-the-art overview of the different components of the dental pellicle is missing. Due to the advancement in analytical techniques for studying nanoscale objects and processes, there has been an increasing number of new papers in the field of pellicle research, especially in recent years. Therefore, the aim of this review article is to give an updated overview on the composition of the dental pellicle and to identify future research areas.

## 2. Materials and methods

For this review, the PubMed-database was searched using the following search-terms: “pellicle AND (teeth OR tooth OR enamel OR dentin) AND composition”. The literature search was completed on March 31, 2023. Relevant studies were selected through independent review by JE and FM. Studies with a general focus on the composition of the dental pellicle were included. Exclusion criteria were studies where the pellicle was investigated on restorative materials, or where the effect of different external ingredients on the pellicle was investigated. Studies published before 1990 were excluded.

### 2.1. Analytical techniques

Several analytical techniques can be used for the research on the dental pellicle. The techniques are presented in short in Table 1. Techniques, after collecting the samples, are used to 1. separate the fractions, and 2. analyze the composition of the respective fractions.

**Table 1:** Analytical techniques, and short description on the methodology.

Analytical technique	Description
*ESI—Electrospray Ionization*	Electrospray ionization is used in combination with mass spectrometry. This method is used to separate samples into smaller units that will used for mass spectrometry.
*HPLC—High Pressure Liquid Chromatography*	High pressure liquid chromatography is used to separate liquids into smaller units using high pressure. The smaller fractions can then be used for further analysis, such as mass spectrometry.
*LC- Liquid Chromatography*	Liquid chromatography is used to separate liquids into their components. In contrast to HPLC, (porous) membranes will be used instead of high pressure.
*MS—Mass spectroscopy*	Mass spectrometry detects particles based on their mass. After detection of the particles, the spectra can be compared with databases. Following this, molecules can be assigned.
*MALDI—Matrix-assisted laser desorption*	Matrix assisted laser desorption is used for the separation of samples into smaller particles.
*SDS-PAGE—Sodium Dodecyl Sulfate—PolyAcrylamid Gel Electrophoresis*	SDS-PAGE is a technique where proteins are separated based on their respective size in a gel. Following this, fractions can be further analyzed with additional techniques.
*TOF—Time of flight*	The separated ions and molecules are analyzed based on their time of flight. The techniques reveals details on the size of the molecules. Comparing to a database, the composition can be determined.

## 3. Results

**Figure 1:**
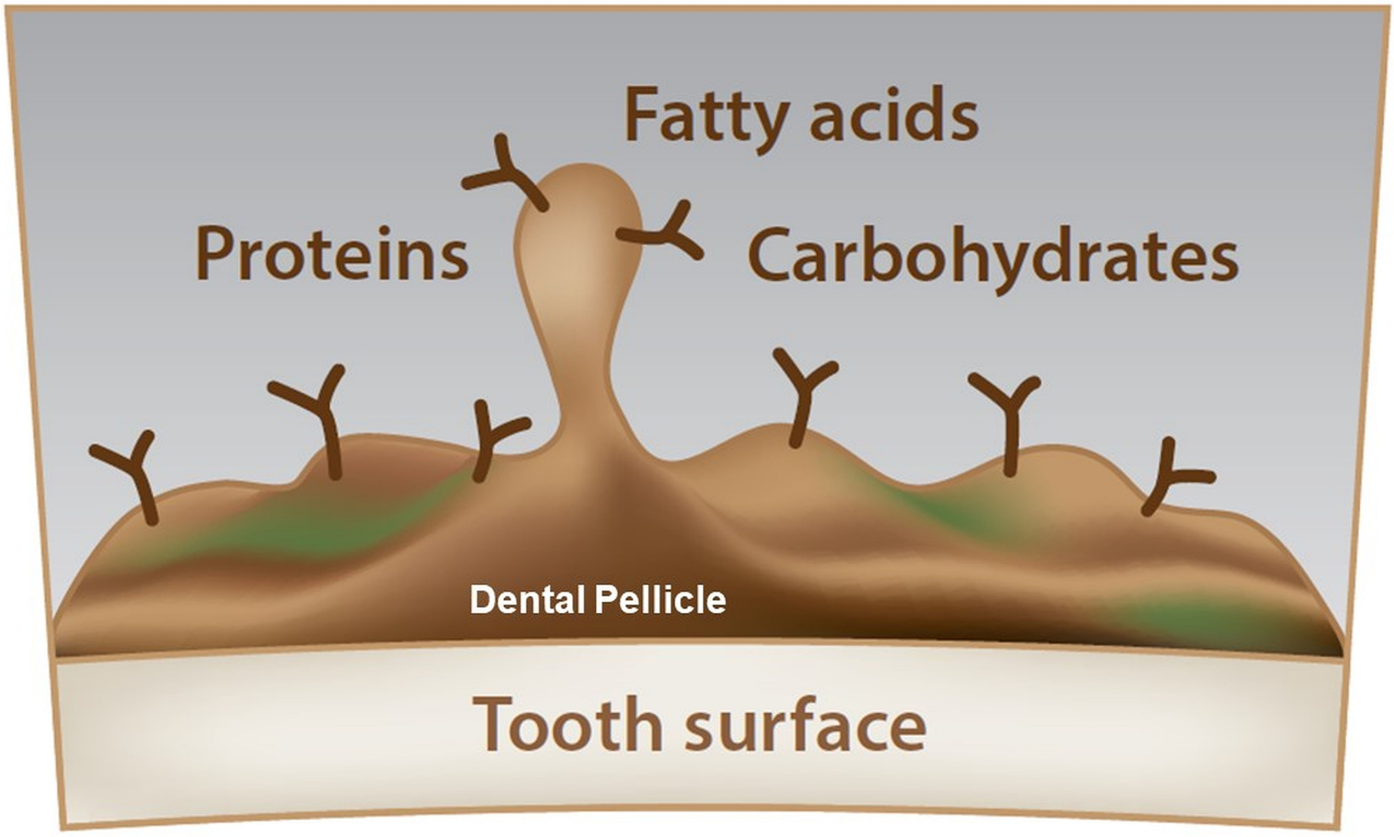
Schematic overview of the dental pellicle. Due to the high complexity, carbohydrates, fatty acids, and proteins cannot be located. The dental pellicle is a thin layer, covering the tooth surface while being the starting point for the bacterial adhesion and biofilm buildup.

The composition of the dental pellicle can be described as highly diverse. The pellicle contains carbohydrates, fatty acids and proteins (Figure 1). Sources of the same are not only saliva, but also diet and also of microbial origin. The included studies can be separated into studies focusing on the carbohydrates, fatty acids and proteins. Table 2 gives an overview of the compounds that have been previously identified in the pellicle.

**Table 2:** Constituents of the dental pellicle and their functions (alphabetical order of proteins and other components).

Study/author	Constituent	Function	Analytical technique
Amino acids			
Sønju Clasen et al. (32), Rykke et al. (33), Rykke and Sønju (34)	Asparagine Threonine Serine Glutamine Proline Glycine Alanine Valine Methionine Cysteine Isoleucine Leucine Tyrosine Phenylalanine Histidine Lysine Arginine	Parts of proteins/pellicle formation	LC
Carbohydrates			
Chawhuaveang et al. (19)	Carbohydrates: Fucose, galactose, galactosamine, lactose, glucosamine, glucose, mannose, rhamnose	Nutrients for the biofilm	Literature Review
Lipids			
Chawhuaveang et al. (19), Reich et al. (35)	Lipids: Cholesterol, cholesterol esters, glycerides, phosphatidylcholine, lyso-phosphatidylcholines, sphingomyelin, phosphatidylethanolamine, phosphatidylinositols, phospholipids	Acid resistance, ultrastructure	Literature Review, HPLC- MS/MS
Proteins			
Chawhuaveang et al. (19), Odanaka et al. (36), PelA et al. (37), Yao et al. (38), Vitorino et al. (39), Zimmermann et al. (40)	Acidic proline-rich proteins	Lubrication, maintenance of mineral homeostasis, acid resistance	Literature Review, NanoLC-ESI-MS/MS, SDS-PAGE/Western-blot, MALDI-TOF-MS, HPLC
Chawhuaveang et al. (19), Odanaka et al. (36), Rasputnis et al. (15), Siqueira et al. (6), Siqueira et al. (3), Yao et al. (38)	Albumin	Salivary component, immune response, protein-protein interaction	Review of the existing literature, SDS-PAGE/Western-blot, MALDI-TOF-MS, LC-ESI-MS/MS
Chawhuaveang et al. (19), Lee et al. (41), Odanaka et al. (36), PelA et al. (37), Rasputnis et al. (15), Siqueira et al. (4), Siqueira et al. (3), Yao et al. (38), Zimmermann et al. (40)	Alpha-Amylase	Salivary component, antibacterial properties, protein-protein interaction, Ca^2+^ binding	Review of the existing literature, NanoLC-ESI-MS/MS, SDS-PAGE/Western-blot, MALDI-TOF-MS, LC-ESI-MS/MS
Lee et al. (41), Odanaka et al. (36)	Alpha-enolase	Ca^2+^ and PO_4_^3−^ binding	SDS-PAGE/Western-blot, LC-ESI-MS/MS
Lee et al. (41), Siqueira et al. (4), Vitorino et al. (39)	Calgranulin	Calcium binding properties	LC-ESI-MS/MS, HPLC
Lee et al. (41), Zimmermann et al. (40)	Calmodulin	Ca^2+^ binding	LC-ESI-MS/MS, HPLC
Siqueira et al. (4), Siqueira et al. (3)	Carbonic anhydrase	pH-regulation	LC-ESI-MS/MS
Chawhuaveang et al. (19), Lee et al. (41)	Carbonic anhydrase I, II, III	Acid resistance, Ca^2+^ and PO_4_ binding	Review of the existing literature, LC-ESI-MS/MS
Chawhuaveang et al. (19), Odanaka et al. (36), PelA et al. (37), Siqueira et al. (6), Siqueira et al. (3), Vitorino et al. (39), Yao et al. (38), Zimmermann et al. (40)	Cystatin	Antimicrobial properties, acid resistance	Review of the existing literature, NanoLC-ESI-MS/MS, SDS-PAGE/Western-blot, MALDI-TOF-MS, LC-ESI-MS/MS, HPLC
Siqueira et al. (3)	Defensin (neutrophil)	Protein-protein interaction	Review of the existing literature
PelA et al. (37)	Enamelin	Enamel protein	NanoLC-ESI-MS/MS
Chawhuaveang et al. (19), Siqueira et al. (4)	Fibrinogen	Immune response	Review of the existing literature, LC-ESI-MS/MS
Chawhuaveang et al. (19)	Fibronectin	Immune response	Review of the existing literature
Chawhuaveang et al. (19), Lee et al. (41), Vitorino et al. (39)	Histatins	Maintenance of mineral homeostasis, acid resistance	Review of the existing literature, HPLC
Lee et al. (41), Odanaka et al. (36), Zimmermann et al. (40)	Histone	Protein-protein interaction, Ca^2+^	SDS-PAGE/Western-blot, LC-ESI-MS/MS
Chawhuaveang et al. (19), Odanaka et al. (36), PelA et al. (37), Siqueira et al. (4), Siqueira et al. (3)	Immunoglubins (sIgA, IgG, Ig*α* I and II)	Antimicrobial properties, immune response	Review of the existing literature, NanoLC-ESI-MS/MS, SDS-PAGE/Western-blot, LC-ESI-MS/MS
Lee et al. (41), Siqueira et al. (4)	Keratin	Protein-protein interaction, Ca^2+^ and PO_4_ binding	LC-ESI-MS/MS
Chawhuaveang et al. (19), Zimmermann et al. (40)	Lactoferrin	Antimicrobial properties, protein-protein binding	Review of the existing literature, LC-ESI-MS/MS
Lee et al. (41), Zimmermann et al. (40)	Lactoperoxidase	Protein-protein interaction	LC-ESI-MS/MS
Odanaka et al. 2020 (36), Siqueira et al. (4), Yao et al. (38)	Lactotransferrin	Antibacterial properties, protein-protein binding	SDS-PAGE/Western-blot, MALDI-TOF-MS, LC-ESI-MS/MS
Chawhuaveang et al. (19), Lee et al. (41), PelA et al. (37), Odanaka et al. (36), Rasputnis et al. (15), Siqueira et al. (3), Yao et al. (38), Vitorino et al. (39)	Lysozyme	Lysis of cell-walls, antimicrobial properties, Ca^2+^ binding	Review of the existing literature, NanoLC-ESI-MS/MS, SDS-PAGE/Western-blot, MALDI-TOF-MS, HPLC, LC-ESI-MS/MS
Chawhuaveang et al. (19), Lee et al. (41), PelA et al. (41), Rasputnis et al. (15), Siqueira et al. (4)	Mucins (MG1, MG2, 5CB, C7)	Anti-erosive properties, Lubrication, Protein-protein interaction	Review of the existing literature, NanoLC-ESI-MS/MS, LC-ESI-MS/MS
Chawhuaveang et al. (19), Odanaka et al. (36), Yao et al. (38), Zimmermann et al. (40)	Myeloperoxidase/peroxidase	Antimicrobial and antifungal properties	Review of the existing literature, SDS-PAGE/Western-blot, MALDI-TOF-MS, LC-ESI-MS/MS
Odanaka et al. (36)	Serotransferrin	Antibacterial properties	SDS-PAGE/Western-blot
Chawhuaveang et al. (19), PelA et al. (37), Vitorino et al. (39), Yao et al. (38)	Statherins	Lubrication, maintenance of mineral homeostasis, acid resistance	Review of the existing literature, NanoLC-ESI-MS/MS, MALDI-TOF-MS, HPLC

ESI, electrospray ionization, HPLC, high pressure liquid chromatography; LC, liquid chromatography; MS, mass spectroscopy; MALDI, matrix-assisted laser desorption; SDS-PAGE, sodium dodecyl sulfate, PolyAcrylamid gel electrophoresis; TOF, time of flight.

### 3.1. Carbohydrates

Carbohydrates of the dental pellicle are mainly derived from diet and microorganisms. However, the composition of carbohydrates is not well-studied. The main function of carbohydrates is as nutritional source for oral bacteria and the biofilm (19).

### 3.2. Fatty acids

Research on fatty acids in the whole dental pellicle is not well-established in the literature and limited to the characterization of fatty acid profiles of the dental pellicle. Triacylglycerols and phospholipids were found as major compounds. In contrast to this, glycolipids, cholesterol, and cholesterol esters were not found in the pellicle, and saliva (35, 42). Fatty acid profiles (types of fatty acids) of different subjects are comparable. An increase in the total amount of fatty acids can be found over time. However, the total amount of fatty acids is differing between individuals. On the basis of fatty acids, pellicle formation is a selective process, which is not directly correlating with the salivary profile (42).

### 3.3. Proteins

Characteristics of studies focusing on general composition give an overview of the pellicle composition. Rasputnis et al. found that the ultrastructure of the pellicle on dentin and enamel surfaces is comparable. However, as mostly enamel pellicle has been studied, a final statement on the comparability of the composition and properties of the dentin and enamel pellicle cannot be made (15). Chawhuaveang et al. describe the protective properties of the dental pellicle and gave additional insights into its calcium-binding properties that help protect teeth from demineralization. Another important aspect of the pellicle is serving as the binding site of bacteria to the teeth. With this, the pellicle also contributes to periodontal diseases, as the bacterial biofilm adhering to the teeth is a key-factor for periodontal infections. Insights on pellicle’s selective acid-transportation, which determines its caries-protective properties was given (19). It is important to note that the methodology of the pellicle collection influences the composition (3, 37). While former studies and their respective results are based on a gel-based approach (for protein-composition), more recent studies used gel-based techniques combined with mass spectrometry (MS) or immunologic methods (36, 37, 43–45). Odanaka et al, in addition, have investigated the origin of the proteins found in the pellicle. They are not only derived from salivary glands and saliva, but also from gingival crevicular fluid (36). Another factor influencing pellicle composition seems to be the location of sampling: Upper or lower jaw, anterior or posterior teeth, palatal, lingual or facial (46).

When investigating the dental pellicle, it is not only the location that plays an important role, but also the time point of sampling. The pellicle is fully formed after 120 min but is changing with a high dynamic in composition over time (41). Siqueira et al. found 130 proteins in all samples that were extracted from 3 individuals (4). The number of proteins found in the various studies differs from the respective methods that were used. Statherin, lysozyme, albumin, and amylase were intact proteins found in the study by Yao et al. (38). Interestingly, albumin can be found when sampled *in vivo*, but when the same saliva was formed *in vitro* on hydroxyapatite discs, albumin was observed in smaller amounts (47). Histatins, as protective proteins in the oral cavity, can also be found in the pellicle (5). Primary teeth and permanent teeth have a distinct pellicle characteristics: Pellicle from primary teeth forms slower and is thinner, without a globular second layer compared to the pellicle formed on teeth in the permanent dentition (32).

It seems that proteins are also denatured over time. Zimmermann et al. looked at the proteome and peptidome. It can be assumed that the peptidome is derived from the proteome. A clinical trial has shown a unique composition among all subjects (40). Mucins are also known to be part of the pellicle. Levels of mucins are impaired by hyposalivation and reduction of salivary flow (48). The proteolytic activity from different enzymes in the oral cavity seems to influence the formation and composition of the pellicle (39). Proteolysis of the salivary proteins for the formation of the pellicle does not seem to be a random process (49). Proteins differ between sampling sites in the oral cavity. Parotid saliva agglutinin, which is the main binding-site for *Streptococcus mutans*, was identified in the premolar region of the oral cavity (47). Glycosyltransferase, which is known as virulence factor for *streptococci,* can be identified in the pellicle (8). Diet, mainly sugar intake (amount and frequency) and possibly also bacterial biofilms have an influence on the pellicle formation and also on the protein degradation of the same (33, 34). In a 2-year *in vivo* study, Rykke et al. showed different amino acid profiles from saliva and the pellicle indicating that pellicle formation is a selective process (33).

## 4. Discussion

Several studies have investigated the different components of the dental pellicle (3, 19, 35, 48). However, most studies have been performed several years ago using different molecular technologies. These methods include the most commonly used technique for the identification of proteins, namely western blot (21, 36, 47, 50–52). More sophisticated approaches, such as liquid chromatography (LC) combined with mass spectroscopy (MS), or high-pressure-liquid-chromatography (HPLC) were also used alone or even in combinations (4, 9, 20, 35–41, 53). From those approaches, the composition was found to be more complex compared to that found when using western blot alone. Furthermore, up-to-date technologies for proteomics will probably lead to a deeper insight into the dental pellicle. Another important aspect is the integration of microscopic techniques for the characterization of the dental pellicle. Atomic force microscopy is one of those techniques that might help getting more insights into the dental pellicle-characteristics (54). Additionally, lab-research could provide more information on the function and interaction of the identified proteins. Future research needs also to focus on the variation in pellicle composition with sampling sites, ethnicity, age, gender, known systemic diseases, and others. These differences in composition of pellicle from sampling sites might be attributed to the variation in the composition of the saliva, including the proteins, from different salivary glands (36, 46).

It is established that all known amino acids are present in the dental pellicle (33, 34). The ratio of those varies between the studies indicating individual profiles of the proteins. The most often identified proteins in the pellicle are amylase, lysozyme, statherins, mucins, immunoglobulins, peroxidase, cystatins, albumin, and proline-rich proteins (Table 2). Most proteins have the ability to bind Ca^2+^, leading to the assumption that the pellicle plays a protective role against tooth demineralization (3, 19, 36). The pellicle can be a reservoir for calcium ions protecting the teeth from attack by acid from bacteria leading to dental caries or from other sources leading dental erosion. Therefore, it is important not to inactivate the Ca^2+^ but supplement the pellicle. Studies have shown the benefits of adding Ca^2+^ for oral health, especially protecting teeth from demineralization (55–58).

Compared to the studies investigating proteins, fewer studies have been published on the presence and characterization of lipids or carbohydrates in the dental pellicle (3, 19, 20, 35, 42). There is limited evidence that the pellicle might also be a reservoir for carbohydrates (i.e., sugars). While some of those sugars (e.g., xylitol) might be protective, others might enhance the caries-process, namely glucose or lactose (59, 60). It can be assumed that carbohydrates content of the pellicle are mainly derived from diet and will be metabolized by oral bacteria for their energy supply. Lipids might improve the acid resistance of the pellicle and change the microstructure of the pellicle (19, 35, 42, 61).

This review gives an overview of the pellicle composition. However, the structure and interaction of individual pellicle constituents have not been investigated so far. From a biological point of view, the interaction of components within the pellicle and how these interactions may affect its three-dimensional structure is important, as this might help to understand how the pellicle can be modified to protect the teeth from acids and bacterial adhesion. By reducing bacterial adhesion, risk for dental caries and gum diseases can be reduced (62–64). Interestingly, calcium phosphates lead to a reduction of biofilm formation. Hydroxyapatite particles, which have been shown to prevent dental caries in several clinical trials (65–67), seem to reduce the biofilm formation to a higher extend compared to amorphous calcium phosphates in combination with casein phosphopeptides (62, 68).

From a scientific point of view, the dental pellicle needs to be characterized more thoroughly in a variety of ways. Those include a detailed description of its three-dimensional structure, the interaction of proteins in the pellicle, the interaction of the pellicle with bacteria and fungi during biofilm formation, but also the role of carbohydrates and lipids. While many proteins, carbohydrates and others have been identified as part of the pellicle, their involvement in the remineralization process remains mostly unclear. Casein has been identified as important calcium-binding protein, and others also have calcium-binding abilities, but their specific roles in remineralization need further investigation. Additionally, proteins might penetrate small cracks and demineralized structures, where they could bind calcium and thus enhance remineralization in those non-intact areas.

Future research needs to focus not only on these questions, but also on the modification of the pellicle. Studies have shown that different active agents can be incorporated into the pellicle or alter the biofilm adhesion. Those are hydroxyapatite particles, but also polyphenols (12, 62, 69–72). It is important to keep the oral homeostasis intact and to achieve this, biomimetic approaches are preferred (73–75). This means that biomimetic remineralization using calcium phosphates should be enhanced when modifying the pellicle (57, 58). Additionally, bacterial adhesion needs to be reduced and acidic resistance needs to be enhanced (56, 76). While the pellicle is known to show subtle variations in structure and composition between individuals, there may be beneficial modifications to the pellicle that can be applied uniformly to every single individual with consistent results. This, however, requires further investigation.

## 5. Conclusions

This review has summarized the composition of dental pellicle. While many proteins have been identified so far, further research needs to be performed especially on the intra-individual composition and the function of the proteins, lipids, and carbohydrates. Furthermore, modification of the pellicle to enhance its beneficial functions is possible, mainly with effective agents to protect teeth from acidic attacks or to inhibit bacterial adhesion. The research focus needs to be based on active biomimetics as they will be positively influencing oral homeostasis. In addition, future research using the most advanced analytical methods needs to focus on the involvement of the dental pellicle in the remineralization process.
